# Spatial calibration of a 2D/3D ultrasound using a tracked needle

**DOI:** 10.1007/s11548-016-1392-5

**Published:** 2016-04-08

**Authors:** Francisco Vasconcelos, Donald Peebles, Sebastien Ourselin, Danail Stoyanov

**Affiliations:** Centre for Medical Image Computing, UCL, London, UK; Department of Obstetrics and Gynecology, UCL, London, UK

**Keywords:** 3D Ultrasound, Calibration, Instrument Tracking

## Abstract

**Purpose:**

Spatial calibration between a 2D/3D ultrasound and a pose tracking system requires a complex and time-consuming procedure. Simplifying this procedure without compromising the calibration accuracy is still a challenging problem.

**Method:**

We propose a new calibration method for both 2D and 3D ultrasound probes that involves scanning an arbitrary region of a tracked needle in different poses. This approach is easier to perform than most alternative methods that require a precise alignment between US scans and a calibration phantom.

**Results:**

Our calibration method provides an average accuracy of 2.49 mm for a 2D US probe with 107 mm scanning depth, and an average accuracy of 2.39 mm for a 3D US with 107 mm scanning depth.

**Conclusion:**

Our method proposes a unified calibration framework for 2D and 3D probes using the same phantom object, work-flow, and algorithm. Our method significantly improves the accuracy of needle-based methods for 2D US probes as well as extends its use for 3D US probes.

## Introduction

Ultrasound (US) is widely used in medical diagnostics and during therapy as a low-cost, flexible, and real-time imaging technique. Particularly in fetal medicine, US is both used for noninvasive diagnostics and to guide surgical interventions, such as biopsies. 3D ultrasound devices (3D US) introduce a new range of applications in this domain by enabling the acquisition of real-time 3D volumes and 4D video.

**Fig. 1:**
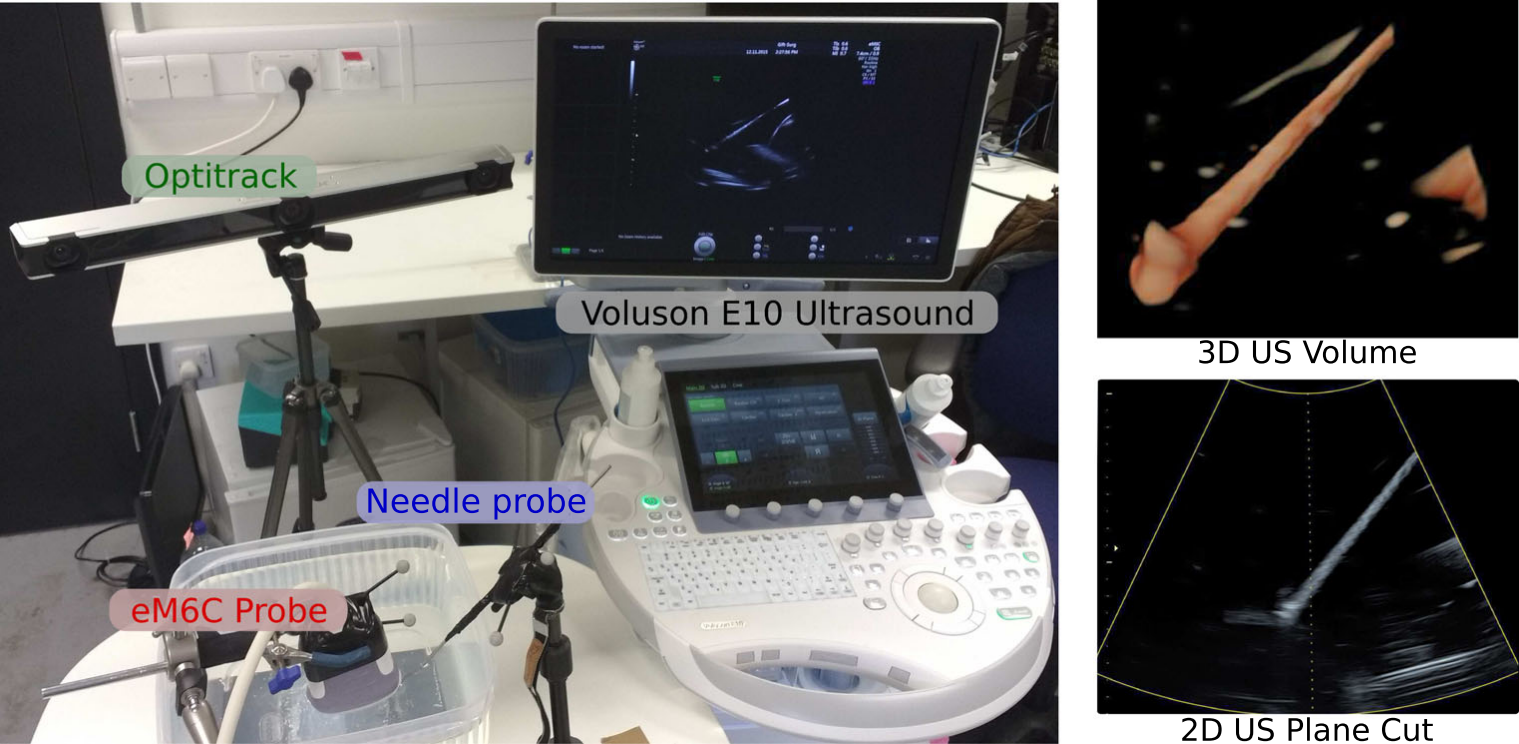
3D US calibration system: GE Voluson E10 with eM6C probe, Optitrack V120 Trio, and a tracked needle. Example of 3D volume and 2D slice showing the appearance of a needle in the US field of view

Pose tracking and calibration of a 3D US probe enhances its applications to computer-assisted intervention (CAI), in order to transfer the information from US scans to other coordinate frames. Large and detailed 3D models can be built from several 3D US frames [6, 25] using pose tracking instead of 3D model registration. A tracked 3D US probe enables freehand 4D US [11], i.e., registering both a 3D volume and its temporal evolution in a single coordinate system while the probe is being freely moved. Pose tracking also allows registration between 3D US data and other instruments, such as biopsy needles, without requiring segmentation on US volumes [24], while the needle or any other instrument with known shape can itself be used as a calibration phantom (Fig. 1).

Although real-time 3D US is a recent technology there has been an extensive study of 3D US imagery using 2D probes (2D US) using either the freehand 3D technique [2] or a motor swept probe [9]. Freehand 3D US is achieved with a 2D US probe attached to a pose tracking sensor. By moving the probe while tracking its pose for every scan acquisition one is able to build a 3D volume from a collection of 2D scans. This is the most widely studied and cost effective approach so far to obtain 3D US imagery but it also has some drawbacks. This approach is only able to reconstruct 3D volumes if the scanned region is static during a probe swipe, and therefore it becomes extremely challenging or inapplicable to dynamic environments such as obstetrics. The effective use of this approach must also be preceded by a non-trivial and time-consuming calibration procedure which is often challenging to export from the laboratory environment to a real medical scenario. A second approach to produce 3D volumes uses a 2D US swept by a controlled motor. This can greatly simplify the calibration procedure; however, in most cases the probe must be fixed during a complete motor swipe, and thus it is not suitable for a freehand scenario. Real-time 3D US can be achieved with probes containing a 2D array of US sensors that enable fully synchronized capture of 3D US volume. Although these probes make it significantly easier to obtain 3D acquisitions, they are still a costly solution.

Spatial calibration between a 2D US and a tracking system is a widely studied topic using a variety of different phantoms[16]. Proposed solutions include scanning a single plane [19, 21, 23], a set of wires, a set of spheres, a stylus/needle [12, 17], and also more complex patterns [18]. Most methods require a very careful placement of the probe in relation to the phantom in order to obtain calibration measurements, making the procedure very time consuming and non-trivial for an inexperienced user. Khamene et. al. [14] proposed a particularly simple calibration procedure that involves scanning any part of a needle under different poses; however, this method is significantly less accurate than alternative methods that require a precise scanning of the needle/stylus tip [12, 17].

There is also previous work on US calibration of swept motor 3D US using scans of a set of wires [5, 15, 20], a stylus tip [20], and a single plane [1, 3, 10]. These calibration methods are particularly interesting since they work with 3D volumes and thus can in principle also be used to calibrate a real-time 3D US. However, the 3D US allows for freehand motion during calibration and therefore allow for more practical calibration procedures. Spatial calibration of a real-time 3D US has only been briefly studied [4, 13] using sets of wires/spheres, single plane, and also more complex multi-object phantoms.

In this paper, we propose a new solution for calibrating both a 2D US and a 3D US using a needle. In a similar fashion to [14] our method allows to scan any part of the needle making it easier to use than most alternative methods. In the case of a 2D US we are able to significantly improve the accuracy of [12, 14]. In the 3D US case we provide the first solution for this type of method. Additionally we propose a unified framework for calibrating both a 2D US and a 3D US, using the same phantom, algorithm, US model, and calibration work-flow. We show promising calibration accuracy results for both the 2D US (2.49 mm mean error for a 107 mm scanning depth) and the 3D US (2.39 mm mean error for a 107 mm scanning depth).

**Fig. 2:**
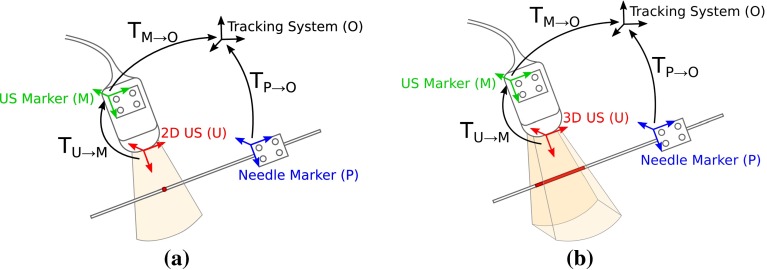
2D/3D Calibration procedure. **a** Schematic of the 2D US calibration problem, where the needle is measured as a point by the US scan; **b** Schematic of the 3D US calibration problem, where the needle is measured as a line segment by the US scan

## Methods

### Calibration procedure

We consider the calibration problem depicted in Fig. 2 composed by a 2D/3D US system and a needle. Both instruments are attached to markers that track their poses in a stationary reference frame $\mathsf {O}$. The system is able to perform simultaneous acquisition of the US scan, the pose of the US marker $\mathsf {T}_{\mathsf {M} \rightarrow \mathsf {O}}$, and the pose of the needle marker $\mathsf {T}_{\mathsf {P} \rightarrow \mathsf {O}}$. In order to represent the US scans and the needle in the same reference frame, both instruments must be calibrated. Calibrating the needle is achieved by determining two of its points using a standard stylus calibration. Calibrating the US is done by determining the transformation $\mathsf {T}_{\mathsf {P} \rightarrow \mathsf {O}}$ and the scale factors that convert from pixels in the US image to metric coordinates. The proposed calibration method requires to scan an arbitrary region of the needle under different poses using the same workflow as in [14] both for a 2D US or a 3D US.

### 3D/2D us model

A homogeneous point $\mathbf {X}_{i}$ in a 3D US volume is mapped to homogeneous metric coordinates $\mathbf {P}_{i}$ in the reference frame $\mathsf {M}$ of the US attached rigid body by means of a scale transformation $\mathsf {S}$ followed by a rigid transformation $\mathsf {T}_{\mathsf {U} \rightarrow \mathsf {M}}$. The scale transformation $\mathsf {S}$ maps from pixel to metric coordinates and generally has the form

1 $$\begin{aligned} \mathsf {S} = \begin{pmatrix} s_{x} &{}\quad 0 &{}\quad 0 &{}\quad 0 \\ 0 &{}\quad s_{y} &{}\quad 0 &{}\quad 0 \\ 0 &{}\quad 0 &{}\quad s_{z} &{}\quad 0 \\ 0 &{}\quad 0 &{}\quad 0 &{}\quad 1 \end{pmatrix} \end{aligned}$$

The rigid displacement $\mathsf {T}_{\mathsf {U} \rightarrow \mathsf {M}}$ maps from the US reference frame $\mathsf {U}$ to the US marker reference frame $\mathsf {M}$ and consists of a translation $\mathbf {t}$ and a rotation $\mathsf {R}$

2 $$\begin{aligned} \mathsf {T}_{\mathsf {U} \rightarrow \mathsf {M}} = \begin{pmatrix} \mathsf {R} &{}\quad \mathbf {t} \\ 0 &{}\quad 1 \\ \end{pmatrix} \end{aligned}$$

We can finally denote the complete transformation $\mathsf {A} = \mathsf {T}_{\mathsf {U} \rightarrow \mathsf {M}} \mathsf {S}$ such that

3 $$\begin{aligned} \mathbf {P}_{i} = \mathsf {A} \mathbf {X}_{i}, \quad \quad \mathsf {A} = \begin{pmatrix} A_{11} &{}\quad A_{12} &{}\quad A_{13} &{}\quad A_{14} \\ A_{21} &{}\quad A_{22} &{}\quad A_{23} &{}\quad A_{24} \\ A_{31} &{}\quad A_{32} &{}\quad A_{33} &{}\quad A_{34} \\ 0 &{}\quad 0 &{}\quad 0 &{}\quad 1 \end{pmatrix} \end{aligned}$$

Although $\mathsf {A}$ has 12 parameters, it only has 9 degrees of freedom (3 in rotation, 3 in translation, 3 scale factors). In some special cases, e.g., for some curvilinear probes, $\mathsf {A}$ might have a single-scale factor ($s_{x} = s_{y} = s_{z}$) and in this case $\mathsf {A}$ has only 7 degrees of freedom.

Note that this model is also valid for a 2D US probe. We can consider, without loss of generality, that the 2D US image plane is $z=0$, therefore $$\mathbf {X}_{i} = {\begin{pmatrix} x_{i}&y_{i}&0&1 \end{pmatrix}}^\mathsf {T}$$ and we can think of the image points as co-planar points in the 3D space. In this case we also assume that $s_{z} = 0$ and thus the third column of $\mathsf {A}$ is also zero ($A_{13} = A_{23} = A_{33} = 0$).

**Fig. 3:**
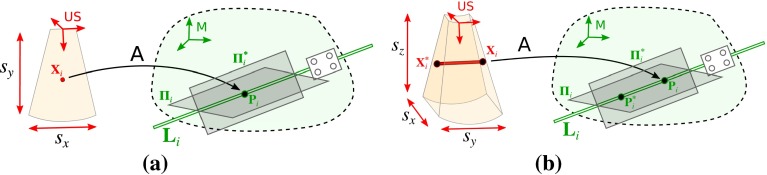
Formulating 2D/3D US calibration as 3D point—3D plane registration. *Each line* $\mathbf {L}_{i}$ can be re-defined as two intersecting planes ${\varvec{\Pi }}_{i}$, ${\varvec{\Pi }}^{*}_{i}$. **a** 2D US. **b** 3D US

### 2D US calibration

In each calibration acquisition the tracking system measures the needle as a 3D line $\mathbf {L}_{i}$ in the reference frame $\mathsf {M}$ and the US scan measures an image point $\mathbf {X}_{i}$. For several acquisitions the 2D US calibration problem becomes the registration between 3D co-planar points and 3D lines. Ramalingam et. al. showed that any 3D registration problem involving 3D planes, lines and/or points can be re-stated as the registration between 3D planes and 3D points [22]. In our calibration problem, this can be easily achieved by defining each line $\mathbf {L}_{i}$ as two intersecting planes ${\varvec{\Pi }}_{i}$, ${\varvec{\Pi }}^{*}_{i}$ (Fig. 3). Given that $\mathbf {P}_{i} = \mathsf {A} \mathbf {X}_{i}$ is contained in both ${\varvec{\Pi }}_{i}$ and ${\varvec{\Pi }}^{*}_{i}$, the constraints for each 2D US acquisition are

4 $$\begin{aligned}&{{\varvec{\Pi }}_{i}}^\mathsf {T}{\mathsf {A}} \mathbf {X}_{i} = 0 \end{aligned}$$

5 $$\begin{aligned}&{{\varvec{\Pi }}^{*}_{i}}^\mathsf {T}{\mathsf {A}} \mathbf {X}_{i} = 0 \end{aligned}$$

For *N* acquisitions a system of 2*N* linear equation is built with the 9 nonzero parameters of $\mathsf {A}$ as unknowns. This can be solved with SVD decomposition.

### 3D US calibration

In the case of a 3D US the measurements of the needle are 3D lines $\mathbf {B}_{i}$ instead of 3D co-planar points $\mathbf {X}_{i}$. Following the strategy proposed in [22], we can define the lines $\mathbf {B}_{i}$ as two 3D points $\mathbf {X}_{i}$, $\mathbf {X}^{*}_{i}$. Therefore, the 3D US calibration problem is also re-stated as the registration between 3D planes and 3D points in the same way as the 2D US problem. However, in this case there are two additional constraints

6 $$\begin{aligned}&{{\varvec{\Pi }}_{i}}^\mathsf {T}{\mathsf {A}} \mathbf {X}^{*}_{i} = 0 \end{aligned}$$

7 $$\begin{aligned}&{{\varvec{\Pi }}^{*}_{i}}^\mathsf {T}{\mathsf {A}} \mathbf {X}^{*}_{i} = 0 \end{aligned}$$

For *N* acquisitions a system of 4*N* linear equations is built with the 12 parameters of $\mathsf {A}$ as unknowns. Note that this system is equivalent to a 2D US calibration with twice the number of acquisitions, where $\mathbf {X}^{*}_{i}$ adds the extra constraints.

### Calibration algorithm pipeline

After the calibration acquisition is performed, the above linear equations are used within a RANSAC robust estimator [8] in order to automatically remove outlier acquisitions and give an initial estimate for $\mathsf {A}$. The transformations $\mathsf {T}_{\mathsf {U} \rightarrow \mathsf {M}}$ and $\mathsf {S}$ can be extracted from $\mathsf {A}$, which has the following format

8 $$\begin{aligned} \mathsf {A} = \begin{pmatrix} \mathsf {A}^{*} &{}\quad \mathbf {t} \\ 0 &{}\quad 1 \end{pmatrix} \end{aligned}$$

where $\mathbf {t}$ is the translation component of $\mathsf {T}_{\mathsf {U} \rightarrow \mathsf {M}}$ and $\mathsf {A}^{*}$ is a $3\times 3$ matrix containing both the rotation component $\mathsf {R}$ of $\mathsf {T}_{\mathsf {U} \rightarrow \mathsf {M}}$ and the 3 scale factors of $\mathsf {S}$. These two components can be extracted with QR decomposition

9 $$\begin{aligned} \mathsf {A}^{*} = \mathsf {R} \mathsf {S}^{*} \end{aligned}$$

with

10 $$\begin{aligned} \mathsf {S}^{*} = \begin{pmatrix} s_{x} &{}\quad 0 &{}\quad 0 \\ 0 &{}\quad s_{y} &{}\quad 0 \\ 0 &{}\quad 0 &{}\quad s_{z} \\ \end{pmatrix} \end{aligned}$$

The QR decomposition guarantees that the rotation component is always an orthonormal matrix; however, with noisy measurements $\mathsf {S}^{*}$ is not a diagonal matrix but upper triangular instead. Therefore, the non-diagonal elements must be forced to zero. If the probe is curvilinear, it is usually more accurate to also force all scale factors to be the same ($s_{x} = s_{y} = s_{z}$). Additionally, if the probe is 2D, $s_{z}$ should be forced to zero.

This initial calibration estimate is then refined using Levenberg–Marquadt iterative optimization. This final step aims at finding the calibration solution with minimum euclidean distance between the 3D lines $\mathbf {L}_{i}$ and the projected 3D points from the US image $\mathbf {P}_{i} = \mathsf {A} \mathbf {X}_{i}$. The refined solution is parameterized by 3 translation parameters ($\mathbf {t}$), 3 rotation parameters ($\mathbf {R}$ is represented as a unit norm quaternion), and either 3 scale factors (3D US) or 2 scale factors (2D US).

For the 2D US the optimization problem is

11 $$\begin{aligned} \min _{\mathsf {R}, \mathbf {t}, s_{x}, s_{y}} \sum _{i = 1}^{N} \left( d(\mathbf {L}_{i},\mathsf {A} \mathbf {X}_{i})^{2} + d(\mathbf {L}^{*}_{i},\mathsf {A} \mathbf {X}_{i})^{2} \right) \end{aligned}$$

and for the 3D US

12 $$\begin{aligned}&\min _{\mathsf {R}, \mathbf {t}, s_{x}, s_{y}, s_{z}} \sum _{i = 1}^{N} \left( d(\mathbf {L}_{i},\mathsf {A} \mathbf {X}_{i})^{2} + d(\mathbf {L}^{*}_{i},\mathsf {A} \mathbf {X}_{i})^{2} \right. \nonumber \\&\quad \left. +\, d(\mathbf {L}_{i},\mathsf {A} \mathbf {X}^{*}_{i})^{2}+ d(\mathbf {L}^{*}_{i},\mathsf {A} \mathbf {X}^{*}_{i})^{2} \right) \end{aligned}$$

where $d(\mathbf {L}_{i},\mathbf {P}_{i})$ represents the Euclidean distance between line $\mathbf {L}_{i}$ and point $\mathbf {P}_{i}$.

## Experiments

Our calibration method is tested using the setup displayed in Fig. 2 that includes a GE Voluson E10 machine with a eM6C probe (3D US) and a 333 mm long metal needle. Both instruments are tracked by the infrared camera system Optitrack V120 Trio, which has sub-millimeter accuracy according to its specifications. Experiments were conducted in a container filled with water at room temperature. In each acquisition both the needle and the US probe were held by clamps, and thus temporal synchronization between the tracking system and the US probe was not required.

**Fig. 4:**
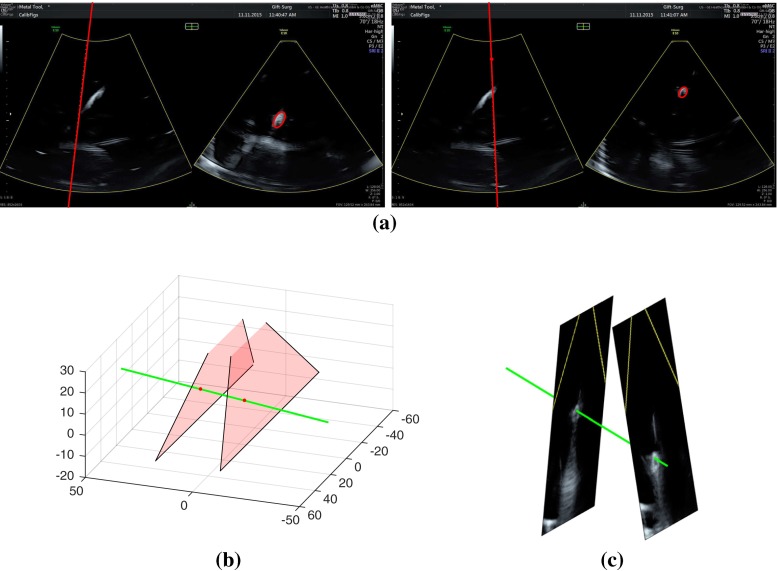
Different plane cuts of the 3D US volume acquisition: **a** 2D/3D US *line segmentation*. Multiple slices of the needle (*red ellipses*) can be generated by changing the angle of the cutting plane (*red lines*). **b** Example with simulated data showing 2 scan planes and the needle. **c** Example with real data showing 2 scan planes and the needle

A sample 3D multi-slice acquisition with the eM6C probe is displayed in Fig. 4a. The image contains two orthogonal scans, whose intersecting axis is represented on the left scan. A section of the needle is detected as an ellipse on right scan. The axis and the needle section are obtained with automatic line and ellipse detectors, respectively, after manually selecting the broad image region where they are located. The center of each ellipse defines a point belonging to the needle. The angle of the intersecting axis can be freely changed, producing multiple scans of the needle for a single instant.

We use the same probe both for 2D US and 3D US acquisition. In our experiment, a 3D US acquisition contains two images with different intersecting axes, each of them containing a cross section of the needle (Fig. 4b) that corresponds in our formulation to one of the points $\mathbf {X}_{i}$, $\mathbf {X}^{*}_{i}$. All 3D US acquisitions are done with the two scan slices forming an angle between 5 and 15 degrees. When tuning this angle there is a trade-off between line orientation accuracy (slices with significant angles between them) and 3D US segmentation accuracy (both slices with close to normal incidence relative to the needle). 2D US acquisitions are obtained by maintaining a constant intersecting axis and using only the information from the right scan of Fig. 4a.

In section “Simulation” we display synthetic results from a simulated environment that reproduces the set-up described above, and in section “Real data” we display results from real data of the eM6C probe. In all cases the calibration procedure is tested for an increasing number *N* of input acquisitions. For each number of acquisitions we perform 20 calibration trials by randomly selecting *N* acquisitions from a total of 30.

### Simulation

The simulated environment contains a 2D/3D US probe with a depth range of 107 mm, angle range [$-$50$^{\circ }$, 50$^{\circ }$], and a single-scale factor $s_{x} = s_{y} = s_{z} = 0.24$ mm/px. The needle is simulated as a line segment with 400 mm length, and is randomly generated at 30 different positions within the field of view of the US. The lines are intersected with a single scan plane (2D US case) or with two scan planes (3D US case) and generate a set of points in pixel coordinates as the US measurements. Both the line locations and the US points are injected with gaussian noise ($\sigma = 2$ pixel, $\sigma =1$mm respectively) in order to simulate measurement errors.

**Fig. 5:**
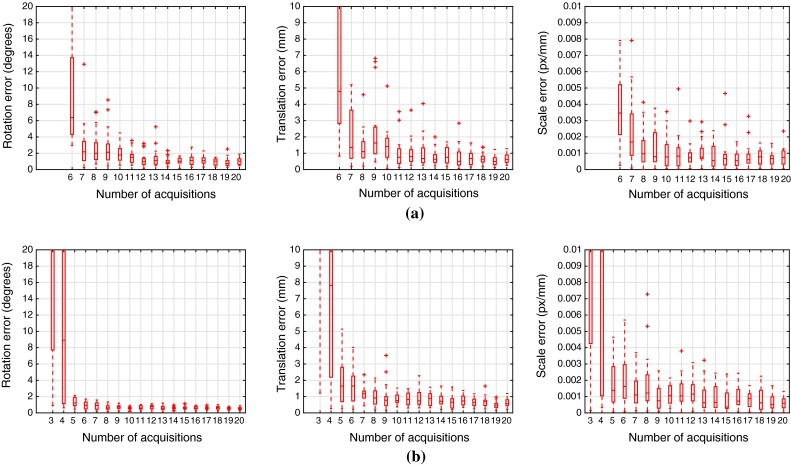
Simulation results for 2D/3D US calibration. Error distribution relative to ground truth values for 20 simulated trials using an increasing number of acquisitions. Results converge at around 10 acquisitions, with the 3D US being consistently more accurate than the 2D US for any number of acquisitions. **a** 2D US. **b** 3D US

**Fig. 6:**
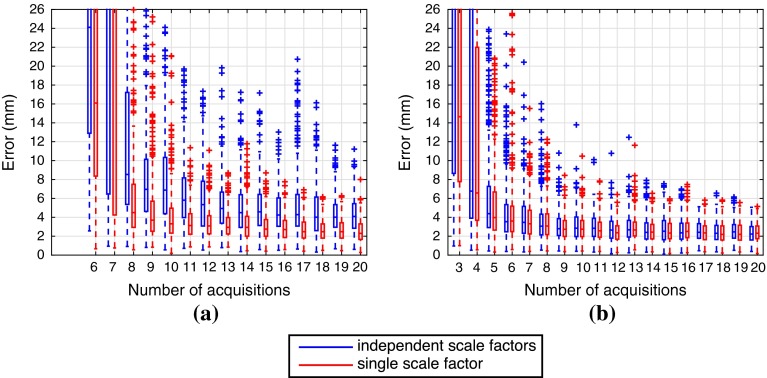
Point reconstruction accuracy on X-shaped wire phantom. Distribution results for (20 calibration trials $\times$ 10 wire phantom acquisitions). The assumption of a single-scale factor ($s_{x} = s_{y} = s_{z}$, in *red*) is significantly more accurate and thus represents a better model of the eM6C probe. Results converge between 10 and 20 acquisitions. **a** 2D US. **b** 3D US

The simulation results are displayed in Fig. 5. We compare calibration trials against ground truth values of rotation ($\mathsf {R}_{GT}$), translation ($\mathbf {t}_{GT}$), and scale factor ($s_{GT}$). The rotation error is measured as the angle displacement of the residual rotation ${\mathsf {R}}^\mathsf {T} \mathsf {R}_{GT}$, the translation error as $||\mathbf {t}_{GT} - \mathbf {t}||$ and the scale error as $|s_{GT} - s|$. The distributions are presented as MATLAB boxplots: the central mark is the median, the box limits are the 25th and 75th percentiles, the whiskers are the maximum and minimum inliers, and individual crosses are outliers.

### Real data

Overall, the parameters in this experimental setup are close to the simulated environment, however there are some notable differences. Unlike in the simulated environment we are not able to directly control the scale factor parameters ($s_{x}$, $s_{y}$, $s_{z}$) and thus we test two possible scenarios: in the first the scale factors are assumed to be different and independent and in the second case we assume a single-scale factor ($s_{x} = s_{y} = s_{z}$) like in the simulated environment. This will affect the number of estimation parameters in the Levenberg–Marquardt optimization step (9 parameters for the first case, 7 for the second case).

To validate the calibration accuracy, we use an x-shaped wire phantom and measure the projection reconstruction accuracy (PRA) of the wire intersection point, i.e., the difference in millimeters between the phantom point location $\mathbf {P}$ measured using the needle and the projection from the US scan $\mathsf {A} \mathbf {X}$. We performed 10 acquisitions of the wire phantom in order to cover different regions of the US scan. Figure 6 displays the distribution of PRA results for all trials. Each distribution contains 200 error measurements (20 trials $\times$ 10 phantom scans).

**Table 1:** 2D US

	This paper	M. & B. 2008 [12]	U. in M. & B. 2015 [19]	U. in M. & B. 2014 [18]
Accuracy (mm)	$2.49 \pm 1.15$	$7.75 \pm 5.04$	$1.3 \pm 1.0$	$0.09 \pm 0.39$
Scan range (mm)	107	30	60	55
Accuracy/range (%)	2.33	25.2	2.1	0.16
Scale factors	Variable	Fixed	Variable	Variable
# of acquisitions	20	20	20	65
Phantom type	Needle	Needle	Single plane	Multi-plane

**Table 2:** 3D US

	This paper	Medical imaging 2013 [13]		
Accuracy (mm)	$2.34 \pm 1.07$	3.44	2.93	2.84
Scan range (mm)	107	Unknown	Unknown	Unknown
Accuracy/range (%)	2.23	Unknown	Unknown	Unknown
Scale factors	Variable	Fixed	Fixed	Fixed
# of acquisitions	20	Unknown	Unknown	Unknown
Phantom type	Needle	Multi-point	Multi-figure	Single plane

## Discussion

Both the simulated and real data suggest that the calibration method converges to a stable solution between 10 and 15 acquisitions using either 2D or 3D US data. For the same number of acquisitions, the 3D US calibration is more accurate than 2D US calibration. This is to be expected since each 3D US acquisition provides four constraints while each 2D US acquisition provides only two.

With real data, we confirm that the 3 scale factors $s_{x}, s_{y}, s_{z}$ are equal while using the eM6C probe in a water bath. The results from Fig. 6 show that for a high number of acquisitions the calibration with 3 independent scale factors (blue) converges to the same solution with a single-scale factor. Note that we could test further if there is any skew distortion by allowing the matrix $\mathsf {S}$ from Eq. 1 to be non-diagonal. This could be done by skipping the projection step described in section “Calibration algorithm pipeline” and allowing $\mathsf {S}$ to be an arbitrary upper triangular matrix. Although this is not displayed in this paper due to space constraints, this method also converges to the format described in Eq. 1 for 20 acquisitions. The single-scale factor is a fair assumption for most curvilinear US probes, since they usually produce scans with unitary aspect ratio. Therefore, our further discussion will only focus on the results provided by this assumption (red distributions).

Our method starts to converge around 10 acquisitions, with the 3D US accuracy increasing slightly faster than the 2D US. The PRA stabilizes at 2.49 mm for the 2D US and at 2.39 mm for the 3D US. Although it is possible to obtain good calibrations with as few as 10 acquisitions, there are cases where the calibration is inaccurate (outliers), which is caused by the random selection process sometimes including many acquisitions without significant motions between them. This can be avoided if the user guarantees that the calibration is performed with a significant variety of needle poses, covering different regions of the US scan. As a general rule, the needle should be moved along different rotation directions between each acquisition, as well as its detection in the US scan should cover different regions of the image.

There are very few calibration methods that report reliable results between 10 and 20 acquisitions regardless of the used phantom. In Tables 1 and 2, we compare our results to the literature on US calibration. This comparison, however, should be taken with caution as these methods use different calibration and validation phantoms, US probes, tracking systems, and in some cases different accuracy metrics.

For the 2D US case, we include the state-of-the-art method that uses the same type of phantom as our method [12] as well as the best performing methods for a relatively low number of acquisitions, using planar-based phantoms [18, 19]. After taking into account the differences in US depth range, our method clearly outperforms the alternative needle-based method. Note as well that the results reported in [12] assume that scale factors are already known before calibration, which makes our method more flexible in practice. The single-plane method is slightly more accurate than ours. The multi-plane method is significantly more accurate, however, results are only reported for 65 acquisitions while all the other methods report results for 20 acquisitions. Overall, our method maintains the usability advantages of needle-based methods described in [12] while decreasing its main drawback (poor accuracy), making it a competitive alternative to planar-based methods.

For the 3D US case, we compare our results with the methods described in [13]. Our absolute performance is better, however, [13] does not provide information about the depth range of the 3D US probe or the number of calibration acquisitions, making it very difficult to draw significant conclusions. Although the results of our method are promising, further research is required to compare it with other 3D US calibration methods described in the literature.

## Conclusions

We presented a new method for 2D/3D US calibration that relies on scanning an arbitrary region of a needle or another straight instrument. This method is easy to perform as it does not require any specific alignment between the US probe and the phantom. In the 2D US case the experiments show that our method improves the current state-of-the-art in needle-based methods, with very similar accuracy to recent single-plane methods. Results reported on 3D US calibration literature are still not detailed enough to establish any strong comparisons between our method and other types of phantom. However, our results indicate that accurate 3D US calibration is possible with 10–20 acquisitions, with more stable results than in the 2D US case. Since all calibrations and validations have been performed in water and without temporal synchronization, our method still needs to be tested on a more realistic set-up. Regarding our geometric formulation, some additional gains in accuracy can be obtained by simultaneously solving the linear system and enforcing the structure of Eq. 1, however, this requires to solve a more complex system of nonlinear equations. Additionally, our formulation is suitable for registration between any combination of plane, line, and/or point correspondences, and thus the same algorithm can be extended to other calibration phantoms with different geometry, which calls for further study.
